# From Optimism to Opportunity: New Perspectives on the Osteological Paradox

**DOI:** 10.1002/ajpa.70331

**Published:** 2026-07-31

**Authors:** Clare McFadden, Amy Anderson, Lucie Biehler‐Gomez, Rachael Brown, Kathryn E. Marklein, Sammuel Sammut, Bronwyn Wyatt, Samantha L. Yaussy

**Affiliations:** ^1^ School of Social Science, University of Queensland Brisbane Australia; ^2^ School of Archaeology and Anthropology The Australian National University Canberra Australia; ^3^ Institute of Behavioral Science, University of Colorado Boulder Boulder Colorado USA; ^4^ Laboratory of Forensic Anthropology and Odontology (LABANOF), Department of Biomedical Sciences for Health University of Milan Milan Italy; ^5^ School of Philosophy The Australian National University Canberra Australia; ^6^ Department of Anthropology University of Louisville Louisville Kentucky USA; ^7^ Center for Archaeology and Cultural Heritage University of Louisville Louisville Kentucky USA; ^8^ Independent Researcher Canberra Australia; ^9^ Department of Sociology and Anthropology James Madison University Harrisonburg Virginia USA

## Abstract

The Osteological Paradox has had an immeasurable impact on bioarchaeology. It has spurred introspection, increased scientific rigor, and encouraged a suite of new and valuable approaches and methods to deal with the challenges it raises. Contrastingly, it has also been perceived as insurmountable, or a problem that simply needs to be acknowledged but not dealt with. In this synthesis, we discuss past and current approaches to the Osteological Paradox, the promise we believe agent‐based modeling holds for future research, and broadly contextualize the Paradox within a philosophically optimistic outlook. Our synthesis represents a range of diverse perspectives from bioarchaeology and philosophy, and seeks to identify a path forward. We conclude that the Osteological Paradox represents a springboard more than a hurdle; one that continues to propel the discipline forward and supports the ongoing integration of new technologies to answer questions about past societies.

## 1 Introduction

The Osteological Paradox (also referred to as the “Paradox” hereafter) has been one of the most impactful and ubiquitous concepts in bioarchaeology since its publication in 1992 (Wood et al. 1992). Wood et al. point out how the very nature of the skeletal record creates challenges for interpreting population health from skeletal samples: namely, archaeologists never get to observe the population dynamics and disease processes that shape the demographics and visible disease profiles of a cemetery, and multiple paths through these demographic and epidemiological processes can produce indistinguishable skeletal data sets, especially given the small sample sizes typical in archaeological contexts. The seminal paper on the Paradox has been cited more than 2500 times according to Google Scholar statistics, and its concerns continue to pervade the discipline today. The Paradox outlines a number of key points responsible for producing a many‐to‐one relationship between a single bioarchaeological data set and the plausible interpretations of health in the once‐living population that it represents: that individuals will have different susceptibility to disease and death (hidden heterogeneity); that at each age, the individuals we study through their skeletal remains are likely to be sicker than the general population (selective mortality); and that populations are neither stationary nor stable, rather they are constantly fluctuating from the combined effects of changes in migration, fertility, and mortality (non‐stationarity). Overwhelmingly, it is hidden heterogeneity and selective mortality that the discipline has struggled with most.

Since its publication, the Paradox has troubled many of the foundational assumptions about how we infer health, disease, and mortality from skeletal remains. While the Paradox did not offer a standard interpretive framework—nor did Wood et al. intend it to—it raised profound conceptual challenges that, if left unaddressed, risk undermining population‐level interpretations. More than two decades later, the discipline continues to grapple with how best to respond, despite several notable publications highlighting the weight of the issue and its influence on paleopathological and paleodemographic studies (Wright and Yoder 2003; DeWitte and Stojanowski 2015; Buikstra et al. 2022).

For reconstructing population health from skeletal samples, perhaps the most troubling implication of hidden heterogeneity and selective mortality is that the meaning of a skeletal response to stress or disease remains open to alternative, contradictory explanations. Skeletal lesions take time to develop, making them indicators of at least short‐term survival in the face of stress or disease. But the absence of skeletal lesions can result from being both frailer or more resilient, or from avoiding exposure to lesion‐causing stressors altogether. The hidden heterogeneity among individuals without skeletal lesions hamstrings attempts to make statements about the health consequences of lesion‐causing stress in any particular time or place. Put simply, it is possible for healthier populations to leave behind sicker looking skeletons. The concerns raised by the Osteological Paradox serve as a persistent caveat on our understanding of the challenges faced by human societies in the past and the changes to human survival, health, and longevity through time and space. As there is no changing the inherently unobservable nature of past events, solutions to the Paradox have been difficult to find. Though there is clearly great value in resolving the Paradox, attempts have been limited by several factors, including: the inability to undertake prospective studies of skeletal lesions at the population level; the inherently unobservable nature of past events; and the complexity of skeletal responses to stress, which may be influenced by a range of intrinsic and extrinsic factors.

The current paper synthesizes the discussions from a 2023 workshop hosted online and at the Australian National University in Canberra, Australia. The workshop was promoted via mailing lists, contacts at a number of universities, student groups, and other association‐based forums. A small number of existing collaborators were invited to present. The workshop brought together an international group of applied bioarchaeologists, theorists in biological anthropology and archaeology, and philosophers to re‐conceptualize the Osteological Paradox and take steps towards unraveling it using agent‐based modeling. Notably, this paper does not seek to directly address or respond to Wood et al. (1992), but rather aligns with the predominant issues facing the discipline (being hidden heterogeneity and selective mortality), the common, pragmatic, and innovative ways bioarchaeologists have sought to deal with these, and suggestions for future research. In sections one and two of this synthetic review we discuss the conceptual frameworks and statistical methods that have sought to acknowledge, circumvent, or resolve some of the issues put forth by the Paradox. Noting the limitations of the current suite of tools, in section three we propose the use of agent‐based modeling as a unique opportunity to “observe” the unobservable and a means of performing experiments to test the extent of bioarchaeology's interpretive challenges. Finally, in section four we explore the Paradox from a philosophical perspective, drawing parallels with the uncertainty faced by many other disciplines and reframing the Paradox optimistically, as a clear call to action for improving the rigor of bioarchaeological methods. While this synthesis presents a range of (sometimes differing) perspectives, holistically we believe the work set out here provides an exciting new phase in our field's decades of grappling with the concerns of the Osteological Paradox, and in the epistemology of the discipline more broadly.

## 2 Conceptual Responses to the Osteological Paradox in Paleopathology: A Review of Non‐Statistical Approaches

Despite being so frequently cited, the Osteological Paradox is often referenced superficially rather than critically engaged with (Buikstra et al. 2022). In many cases, researchers acknowledge the Paradox only in passing or employ qualitative strategies to mitigate its implications without altering their interpretive frameworks. This section examines how the core issues raised by the Paradox, particularly hidden heterogeneity in frailty and selective mortality, have been conceptually addressed in paleopathology and paleodemography, with a focus on non‐statistical approaches. The aim here is to foster a more transparent dialogue about interpretive strategies, not to critique specific studies or researchers. Accordingly, we refrain from naming individual works. The goal is not to question the validity of published research, but to reflect candidly and constructively on how the Osteological Paradox is currently treated in the discipline.

### 2.1 The Tip of the Hat: Strategic Use of Caveats and Interpretive Ambiguity

In the absence of a uniformly applicable methodological solution to the problems of the Osteological Paradox, a common strategy has been the strategic use of caveats, or what might be called a “tip of the hat” to the Paradox.

Researchers often acknowledge the Paradox but nevertheless proceed with analysis and interpretations that rely on traditional methods and intuitive interpretations that assume relatively direct relationships between skeletal stress markers and health or survivorship. Such papers directly name the Osteological Paradox, referring to the original publication, or through brief statements in introductions or discussions, and frame it as a general limitation of bioarchaeological data. These caveats include familiar disclaimers: that skeletal lesions represent survivors of disease; that frailer individuals may have died before skeletal changes developed; or that only a subset of conditions is osteologically visible. This approach is the most common method of engaging with the Paradox in bioarchaeological scholarship (Buikstra et al. 2022).

In some cases, the Osteological Paradox is invoked *post hoc*—after results have already been obtained—to explain contradictory or unexpected findings (DeWitte and Stojanowski 2015: 412). This a posteriori engagement can give the impression of theoretical reflexivity; however, when the Paradox is used primarily to reconcile anomalous results with the overarching narrative of a study, it risks functioning more as a rhetorical device than as a substantive analytical framework. The Paradox is not invoked nearly as often when results align with intuitive interpretations of the relationship between skeletal stress indicators and survivability. Rather than shaping research design, hypothesis formulation, or interpretive criteria from the outset, these references tend to smooth over interpretive tensions by retroactively invoking the Paradox. Such gestures, or “conceptual afterthoughts,” do little to address the underlying issues of selective mortality and hidden heterogeneity. While they reflect a superficial awareness of the Osteological Paradox, they rarely engage with its implications in a way that meaningfully alters the structure or logic of the research (Buikstra et al. 2022: 78) by incorporating its premises into hypothesis formulation, sampling logic, and interpretive criteria; for instance, anticipating non‐linear relationships between skeletal lesions and mortality risk, or structuring comparisons around expected heterogeneity in frailty rather than assuming uniform susceptibility.

Ultimately, while these strategic acknowledgments do recognize the influence of the Paradox, they rarely address the interpretive vulnerabilities it exposes. As such, interpretations based on traditional methods remain susceptible to the biases the Paradox so clearly exposed. Moreover, such glancing approaches likely reflect broader pessimistic views of the Osteological Paradox: that it is an insurmountable theoretical hurdle that undermines paleopathological and paleoepidemiological research, but one that must be offered obeisance, nonetheless.

### 2.2 The Workaround: Comparative Framing Without Modeling

Many studies have implicitly, or even explicitly, acknowledged the challenges of the Paradox by shifting from absolute claims about past health to more relative, comparative interpretations. Rather than interpreting lesion frequencies as direct indicators of health status, researchers often reframe their analyses as comparisons across populations or time periods, focusing instead on differences in stress exposure or lived conditions (Siek 2013). This comparative strategy is commonly applied in cross‐population studies—examining variation between sites, cultural groups, or burial contexts—as well as in diachronic analyses that track changes in health indicators over time (DeWitte and Stojanowski 2015; Siek 2013). Importantly, as Wood et al. emphasized, comparative analyses are themselves deeply constrained by selective mortality and population nonstationarity; their continued use reflects disciplinary habit rather than methodological resolution.

This approach represents a conceptual workaround: while it does not resolve the interpretive limitations identified by the Paradox, it represents, rather, a pragmatic strategy that many researchers adopt in an attempt to navigate them, shifting away from attempts to reconstruct population‐wide health status in a direct sense and towards relative comparisons. By focusing on relative differences in summarized group‐level indicators such as estimated sex, age‐at‐death, or inferred social status, these studies aim to produce meaningful insights without relying on problematic assumptions about representativeness or cause‐and‐effect relationships. In this sense, the Osteological Paradox is acknowledged and partially integrated into the research design, even if not directly addressed.

However, this strategy still leaves unresolved many of the issues at the heart of the Paradox. Comparative analyses remain susceptible to the same biases if the groups being compared differ systematically in unobservable ways (Wyatt et al. 2022; Zuckerman 2020). Without formally modeling or otherwise quantifying such differences, even relative claims rest on unstable interpretive ground.

### 2.3 Osteobiography and Individual‐Centered Approaches

Until recently, osteobiographical approaches were often undervalued, perceived as ancillary to the broader aims of paleopathological research (Hosek and Robb 2019). Yet, by focusing on individual lives rather than population‐level trends, they offer a powerful means of documenting the range, severity, and clinical impact of skeletal lesions. Osteobiographies highlight inter‐individual variability and provide nuanced reconstructions of how disease may have affected a person's life course across multiple dimensions of personal experience such as time, comorbidity, and social context. These individualized narratives help deepen our understanding of the lived experience of illness in the past, and its embodiment, and shift paleopathological inquiry from abstract indicators of morbidity to tangible, human stories.

Osteobiographies are sometimes treated as a conceptual refuge from the Paradox, but this perception can obscure the fact that individual‐centered narratives still depend on survivorship and lesion development. Thus, while they sidestep certain population‐level biases, they cannot fully escape the Paradox's implications. When case studies include relative health assessments of an individual in the context of their community, then issues of survivorship bias and lesion timing may still arise. Yet, without contextualization within a broader population or biocultural framework, osteobiographies risk becoming anecdotal or misleading.

Ultimately, while osteobiographical methods avoid many of the problems inherent to population‐level analyses, they must still engage critically with the conceptual challenges posed by the Paradox (DeWitte and Stojanowski 2015; Pfeiffer 2022). Thoughtful integration of lesion chronology, individual frailty, and archaeological context is essential to ensure that these narratives contribute meaningfully to our understanding of health in the past.

### 2.4 Skeptical or Cautious Approaches: Withholding Inference

A final, though less frequently adopted, approach is one of skeptical restraint. In these rare cases, researchers explicitly acknowledge the interpretive limitations imposed by the Paradox and choose to withhold conclusions altogether. Rather than attempting workarounds or partial integrations, this strategy emphasizes the unresolvability of key biases—particularly hidden heterogeneity and selective mortality—and refrains from drawing inferences about past health or demography (DeWitte and Stojanowski 2015; Garcia‐Putnam 2021). Again, such approaches likely reflect a broadly pessimistic view of the Osteological Paradox and the challenges it presents.

Though this highly conservative stance avoids compounding error through speculative interpretation, it offers little in the way of constructive engagement. It neither addresses the underlying conceptual issues nor proposes alternative pathways forward. Rather, it functions as a form of epistemological caution, not a methodological solution. Though acknowledging the limits of skeletal data is necessary, qualitative pronouncements on the unknowability of the past suspend our state of knowledge rather than advancing it.

### 2.5 The Future of Conceptual Approaches to the Paradox

Several of the conceptual strategies developed in response to the Osteological Paradox, such as comparative framing and osteobiographical analysis, offer useful pathways to paleopathological interpretation. However, these approaches can only partially mitigate the issues raised by Wood et al. (1992). Buikstra et al. (2022: 78) report that a consensus of the participants of the 2020 workshop was that “many scholars do not engage with the osteological paradox because they are unsure how to do so, particularly in cases where they face limitations such as fragmentary skeletal remains, poor age estimates, or lack of good chronological control.” One such limitation deserving further attention is the challenge of working with commingled remains, where the analytical unit is no longer the individual, and it becomes exceedingly difficult to assess frailty, survivorship, or lesion patterning meaningfully (Glencross 2014). How the paradox can be addressed in such contexts remains an open question, albeit one that researchers are attempting to grapple with (e.g., Garcia‐Putnam 2021; McCool et al. 2021). Additionally, DeWitte (2009; 2010) has previously presented and discussed multiple interpretations of health and frailty that have been consistent with the data observed in bioarchaeological analyses as a means of contending with the interpretive issues posed by the Paradox.

While conceptual approaches to the Paradox have been the most widely represented means of dealing with (or circumventing) its issues, in parallel there have been a growing number of statistical methods incorporated into bioarchaeological research. These methods have proven highly valuable in their ability to draw more robust inferences regarding health, frailty, and mortality from skeletal remains, and therefore offer another way of dealing with some of the concerns of the Paradox.

## 3 Quantitative Approaches to the Osteological Paradox

### 3.1 Frailty and Risk of Mortality

The term “frailty” was first introduced to bioarchaeology through its use in demographic scholarship, when Vaupel et al. defined heterogeneous frailty as intra‐populational variation in the age‐standardized relative risk of death (Vaupel et al. 1979; Vaupel and Yashin 1985). In their discussion of the Osteological Paradox, Wood et al. (1992) characterized heterogeneous frailty (i.e., heterogeneity in frailty) as a necessary consideration in bioarchaeological studies, as selective mortality acts on existing variation in frailty. In other words, frailty—which is itself influenced by a variety of genetic, biological, environmental, and cultural factors—in turn influences who is “selected” or removed from the population via mortality at different ages. Consequently, the archaeologically‐derived skeletal assemblages studied by bioarchaeologists are inherently biased, as individuals with the highest frailty are disproportionately likely to enter the sample at a given age, whereas their peers with lower frailty are more likely to survive and die at later ages.

Bioarchaeological investigations of heterogeneity in frailty and selective mortality using demographic or epidemiological approaches have broadly concentrated in two areas: (1) studies of the relationships between archaeologically‐ or skeletally‐inferred indicators of frailty and mortality risk or survivorship, and (2) studies of the activity or severity of skeletal lesions and their impact on measures of frailty or resilience. Studies included in the first area often employ hazards analysis or survival analysis to examine the effects of biological or cultural factors on hazards of death or survivorship. In such studies, high frailty is generally characterized by increased risk of death or decreased survivorship, whereas low frailty (resilience) is characterized by decreased risk of death or increased survivorship. For example, O'Donnell and Moes (2021) use Kaplan–Meier survival analysis and a multi‐state model of morbidity and mortality to examine the associations between the presence of enamel hypoplasia on the canine teeth and survivorship and relative risk of death in Ancestral Puebloans. Their results indicate that individuals with enamel hypoplasia had higher hazards of mortality and lower probabilities of survival (O'Donnell and Moes 2021), which is consistent with much of the previous bioarchaeological research on the effects of early life stress on frailty in later life (e.g., Ham et al. 2021).

Although the primary goal of some studies is to understand how the presence of a skeletal indicator or condition influenced frailty in a past population, many studies combine skeletal data and information from the historical or archaeological records to investigate how sociocultural and environmental factors affected patterns of frailty in a given context. For instance, bioarchaeological research has examined variation in survivorship and risk of mortality related to famine (Godde et al. 2025; DeWitte and Yaussy 2020; Yaussy et al. 2016), epidemic disease (Kelmelis and DeWitte 2021; Wissler and DeWitte 2023), temporal periods or cultural transitions (Biehler‐Gomez, Yaussy, et al. 2025; Yaussy et al. 2023), sex or gender (Biehler‐Gomez et al. 2024; Ham 2025; Simon and Hubbe 2025), ancestry (O'Donnell and Edgar 2021; Redfern et al. 2023), and social position or socioeconomic status (Kelmelis et al. 2017; Wyatt and O'Donnell 2025; Yaussy 2024).

Bioarchaeological studies have also contributed to our understanding of how the severity or activity of skeletal lesions influences heterogeneity in frailty. For example, in a study of periosteal new bone formation in individuals from medieval London, DeWitte (2014) found that individuals with active lesions exhibited lower survivorship and individuals with healed lesions exhibited higher survivorship relative to their peers. Similar results were produced in a study of lesion activity in porous cranial lesions (i.e., cribra orbitalia and porotic hyperostosis), which found that active lesions were associated with lower survivorship and greater mortality risk than healed or absent lesions (O'Donnell 2019). Although scholars have noted that not all skeletal lesions can be suitably subdivided into degrees of activity or severity, it has been repeatedly acknowledged that the activity status of a skeletal lesion may provide additional information on heterogeneity in frailty beyond what can be uncovered through the examination of solely lesion presence or absence (McFadden and Oxenham 2020).

### 3.2 Frailty and Cumulative Phenotype Approaches

Other approaches to the study of frailty in bioarchaeology draw from clinical and gerontological literature and practices, in which frailty is defined as a cumulative and systemic phenotype that often correlates with mortality risk (Fried et al. 2001, 2021; Howlett et al. 2021; Rockwood et al. 2005). Although bioarchaeologists may utilize conceptualizations of frailty applied to living populations, we cannot directly compare frailty in the present with the archaeological past. In living populations, frailty is assessed, often over a patient's lifetime, through bodily function/dysfunction and physical abilities, including, but not limited to, observed wasting, osteopenia and osteoporosis, sarcopenia, mobility issues, and declining endurance (Crews 2005; Fried et al. 2001, 2005, 2021; Walston et al. 2006). In contrast, bioarchaeologists must reconstruct frailty in deceased individuals often hundreds of years after their deaths. To make matters more challenging, the substrate from which frailty is subsequently measured (i.e., the skeleton) is both a reflection of childhood (e.g., teeth) and lifetime experiences, as bone tissue is constantly remodeling in response to intrinsic and extrinsic stimuli; and many health conditions do not manifest on the skeleton or take months or years to present.

Several indexical (i.e., cumulative) approaches to frailty phenotypes in bioarchaeology have been proposed in the last 20 years (Petrosino et al. 2025). The earliest efforts to combine multiple skeletal indicators of stress arose from the Global History of Health Project, with Steckel and colleagues' Health Index (Steckel et al. 2002). This novel index enabled researchers to evaluate “health” (0‐poor health to 100‐best health) based on quality‐adjusted life years for seven skeletal and dentoalveolar conditions. In this regard, the Health Index created a composite of health that attempted to address some of the concerns of the Osteological Paradox by weighting skeletal measures alongside life expectancy. However, few researchers adopted this approach to frailty or health assessment, and others have indicated its problematic usage in fragmentary or incomplete skeletal remains.

The skeletal frailty index (SFI) developed by Marklein and Crews (Marklein et al. 2016; Marklein and Crews 2017) reintroduced the cumulative phenotypic approach to bioarchaeological discussions over a decade after the Health Index. Inspired by frailty indices and allostatic load measures in human biology, the SFI presented a simple quantifiable measure for frailty that could be tailored to specific contexts and research questions. The first SFI applied to a medieval London context utilized 13 metric and nonmetric biomarkers often analyzed separately in bioarchaeological research. Presence, absence, or severity of a condition was associated with high (“1”) or low (“0”) frailty; these scores were summed into a cumulative skeletal frailty score, from 0 (lowest) to 13 (highest frailty). Subsequent iterations of the SFI model included fewer frailty biomarkers to facilitate larger sample sizes and, consequently, representability (Marklein and Crews 2017, 2022; Tuggle et al. 2021). Despite age being factored as a covariate in SFI analyses, later comparisons of SFI values showed that both highest and lowest frailty values were reflective of higher mortality risk in adults (Yaussy et al. 2024). Another shortcoming of the original SFI model was the equal weighting of pathological conditions in the cumulative index score.

Zedda et al. (2021) directly addressed the issue of biomarker weighting in indices through their proposed biological index of frailty (BIF). Utilizing the same 13 biomarkers as the SFI, the BIF improved the index by weighting values based on risk of premature death through logistic regression models. Like the Health Index, the BIF also requires only three observable biomarkers, enabling wider applicability to more fragmentary assemblages, with similar limitations as the Health Index (Petrosino et al. 2025).

### 3.3 Combined Approaches to Exploring Frailty

Recent work in bioarchaeology attests to a growing interest in meaningfully integrating the two major approaches to studying frailty in past populations. In some of these studies, researchers have explored the relationship between cumulative measures of stress and overall risk of death to evaluate frailty at the population level. For example, Godde et al. (2020) generated binary frailty scores to compare mortality hazards for individuals with skeletal or dental evidence of frailty (i.e., at least one LEH, CO, or short femur observed) and individuals exhibiting no skeletal lesions or markers. Their results showed significantly increased hazards for individuals with one or more skeletal markers compared to individuals without skeletal markers considered indicative of frailty. Conversely, in a study of frailty in non‐adults from medieval Ireland, increased numbers of skeletal indicators were associated with increased survivorship (Wyatt et al. 2023). Together, these studies indicate how such combinations of skeletal markers (i.e., indices) may track frailty or resilience depending on sample and context.

Another combined approach developed by Yaussy et al. (2024) expressly considers the challenges posed by the Osteological Paradox and addresses the potential shortcomings of the SFI and BIF. While most indices include a standard suite of conditions, this approach uses survival analysis and hazards analysis to identify which conditions are informative about frailty in a given context. As a result, each index developed by this method only includes those conditions associated with significantly lower survivorship and higher hazards of death. Consequently, the resultant index is solely correlated with risk of mortality (i.e., frailty), whereas the original SFI was shown to reflect both frailty and resilience (Yaussy et al. 2024). Given the development of the method as a site‐specific frailty index, the measures of cumulative frailty produced by this approach may vary between skeletal samples (Biehler‐Gomez, Marklein, et al. 2025; Yaussy et al. 2024). Although this approach may preclude direct inter‐site comparisons of cumulative frailty, the hazards‐based cumulative frailty index intentionally considers the susceptibilities and risk factors that influence heterogeneity in frailty and selective mortality in a given context, making it a potentially valuable tool in the exploration of “local biologies” (Lock 1993). The concept of local biologies refers to the entanglement of the material body in local social processes, practices, and circumstances (Niewöhner and Lock 2018), and it has been meaningfully considered in medical anthropology scholarship (Brotherton and Nguyen 2013; Lock 2017; Lock and Kaufert 2001). In contrast, this concept has been underexplored in skeletal studies (Appleby 2019), perhaps because paleopathologists and bioarchaeologists often employ a standardized suite of skeletal and dentoalveolar conditions when examining frailty in skeletonized individuals (e.g., Marklein 2020). To an extent, this practice implies that a given lesion or set of lesions reflects frailty in a consistent manner across populations. Therefore, interrogating the meaning—frailty or resilience—of conditions within a particular historical and socioecological context (i.e., local biologies) promises more accurate interpretations and understandings of site‐specific health.

The major limitation of statistical approaches to health, disease, and mortality in the past is the nature of skeletal remains. Poor preservation, small sample sizes, and sources of bias often render a sample unsuitable for statistical analyses, although new analytical approaches are beginning to effectively address these issues of missing data (Wissler et al. 2022a, 2022b). In contrast, large, well‐preserved samples and historical/modern collections provide researchers with the opportunity to explore the concerns of the Osteological Paradox in a more statistically robust manner, but these are limited in their availability, and it can be challenging to leverage findings across populations due to widely varying demographic and environmental parameters. Noting this, agent‐based modeling can provide researchers with a digital sandpit to play with these parameters and explore the unobservable processes of the past.

## 4 Observing the Unobservable Through Agent‐Based Modeling

Computer simulations, and agent‐based models (ABM) in particular, have firmly established themselves as a hypothesis‐testing approach and become a niche of research within archaeology in the last two decades (Smith and Choi 2007; Lake 2014; Axtell et al. 2002; Graham 2006; Rogers and Cegielski 2017). Application of agent‐based methods to bioarchaeological research problems, however, has predominantly occurred only in the past several years (Wyatt et al. 2024; Wyatt et al. 2025; Anderson and DeWitte 2026; Galeta and Pankowská 2023) [but see also Sattenspiel et al. 2019], but bioarchaeology's limited use of ABMs is not a product of their limited usefulness for bioarchaeological problems. On the contrary, ABMs have immense potential to engage directly with the underlying causes of the Osteological Paradox to better understand the processes which give rise to observed patterns of mortality and pathology in the archaeological record. We present here an introduction to ABMs and a case for their role in addressing the Osteological Paradox.

ABMs are a class of computational models in which the individual agents (people, cells, viruses, villages, etc.) follow a set of if‐then rules that guide their behavior and interactions within the environment of the model (Schelling 1971). The archaeological record is produced by a complex system of multiple interacting processes, and ABMs are a tool for understanding complex systems. These computer simulations are designed to explore how system‐level or aggregate phenomena such as population‐level trends arise from the actions of individual agents (Lake 2015). They provide a setting in which researchers can run systematic experiments—a scientific approach otherwise largely out of reach for history and the social sciences.

Building a computational model requires the researcher to focus explicitly on the nature of the processes that generate their real‐world data. Analytical modeling approaches, which rely on equations to simulate population dynamics, have occasionally been used in bioarchaeology (e.g., Boldsen et al. 2015; Konigsberg and Buikstra 1995), but analytical models are descriptive rather than generative. It is often the unobserved data‐generating processes themselves—disease, mortality, fertility, and the ways in which social inequalities and environmental variation affect them—that bioarchaeologists are interested in reconstructing from observable skeletal data. ABMs put the focus on the relationship between the generative processes and the data that result from them.

ABMs can therefore tackle the foundations of the paradox by (1) outlining the potential confounding effects of the hidden variables that we know contribute to the Osteological Paradox and (2) directly measuring the range of values that result in indistinguishable bioarchaeological data sets. A systematic program of ABM experiments can establish which hidden variables must indeed be addressed, rather than simply acknowledged, in order to understand observed patterns of mortality; and can determine the range of conditions under which population health can, in fact, be reconstructed with reasonable precision and accuracy from bioarchaeological data.

### 4.1 Hidden Effects and Confounding

Perhaps most critical for tackling the Osteological Paradox, computational models are designed to test the implications of changing the value of one or more variables (parameters). This systematic exploration of parameter values in a model is a form of sensitivity analysis known as a parameter sweep. Parameter sweeps are a requisite part of testing an ABM's structural realism (Grimm et al. 2005), revealing the range of parameter values under which the model generates realistic outcomes, and when the processes built into the model return absurd outputs. In a series of agent‐based simulations, multiple parameters are varied systematically across a range of plausible values in order to test their influence on the outcome of interest. Importantly, each variable (parameter) is tested within the context of other variables, delineating when combinations of parameter values result in interaction effects and produce counterintuitive (or “paradoxical”) outcomes.

Such sensitivity analyses make it possible to estimate the values of important but archaeologically unobservable variables (our “known unknowns”), such as individual endogenous frailty and stress incurred from non‐skeletally manifesting sources (Wyatt et al. 2025), or to explore how varying values for age‐dependent skeletal plasticity and lesion formation dynamics alter the resulting relationship between skeletal lesion presence and age at death (Anderson and DeWitte 2026).

Where the presence of hidden variables is theorized but their nature is not well understood, ABMs can narrow the range of plausible estimates by testing a wide array of theoretical or empirically informed parameter values for these variables. The capacity to integrate information from a variety of empirical and theoretical sources and then “fill in” knowledge gaps with estimations of plausible values allows the potential confounding effects of these variables to be controlled for even in real‐world contexts in which these hidden variables remain unmeasurable. This is useful for conceptual variables such as “frailty” in paleodemographic and paleoepidemiological research (Vaupel et al. 1979; Hougaard 1995).

### 4.2 Equifinality

Equifinality is the crux of the Osteological Paradox—that is, multiple potential pathways through the data‐generating processes can produce the same pattern in the resulting observable data, and some of the potential explanations for the observed data may directly contradict each other. This is frequently reflected in the discussion section of many papers (e.g., DeWitte 2010; Jatautis et al. 2011; Porčić and Nikolić 2016) in which alternative plausible explanations are presented for the observed phenomena, and a narrative is sometimes developed as to why the preferred explanation is more likely than the alternatives. Given the invisibility of many of the causal processes behind the data, the relative plausibility of one hypothesis over another can be extremely difficult to gauge with any degree of certainty.

Researchers working with ABMs are encouraged to build multiple alternative models in order to explore the possibilities for equifinality. This is an important strategy because a model that can replicate the patterns of real‐world data is not necessarily the correct model of that reality. It is possible for a single model to generate similar data sets from multiple combinations of distinct parameter values, and it is also possible for multiple models to generate similar data sets using different generative processes. To fully explore modeling solutions to a bioarchaeological problem, researchers should conduct parameter sweeps across a wide range of values for all model parameters, and they must employ creativity and collaboration to build and test a range of plausible generative models. Integration of information from multiple sources–archaeological, epidemiological, cultural and environmental (for instance)–is possible via ABMs and can facilitate parameter development to test numerous scenarios. As a final caution, note that generative models only test the range of explanatory scenarios that the researcher explicitly constructs. The importance of building multiple alternative models therefore extends beyond mathematical considerations of equifinality; this practice can mitigate the limits and biases of the researcher's perspective. If ABMs are to deliver on their potential as a tool for exploring possible histories, researchers employing ABMs will need to reach beyond their own intuitive explanations and invite cross‐cultural perspectives into their model‐generating frameworks.

Ultimately, the lessons learned from ABMs can be used to conduct sensitivity analyses for hidden variables and to inform the distributions of priors in Bayesian approaches rather than relying on frequentist statistics. Konigsberg and Frankenberg (2013) describe bioarcheologists as “think[ing] like Bayesians but act[ing] like frequentists” in that researchers will acknowledge and discuss the uncertainty inherent in our research, but our methods do not address or incorporate this uncertainty. We propose the use of ABMs here to bridge this gap.

Notwithstanding, uncertainty is something that all sciences must deal with. The Osteological Paradox has positioned uncertainty as a central issue in the bioarchaeology of health. Yet, this kind of interpretive uncertainty is a ubiquitous feature of the historical sciences and other fields that rely primarily on observational data rather than controlled experiments. Exploring the ways in which other disciplines deal with drawing inferences from uncertain evidence expands the tools available for dealing with a paradox that is not, in fact, uniquely osteological.

## 5 A Not‐so‐Unique Problem: Underdetermination and Cause for Optimism

The Osteological Paradox is a particularly strong example of something philosophers of science call the underdetermination of theory by data—where we lack sufficient evidence to decide which of two or more incompatible theories or hypotheses is correct (Lewens 2016). In the case of the Osteological Paradox, Wood et al. (1992) argue that skeletal evidence, the bread and butter of bioarchaeology, underdetermines which of many conflicting competing hypotheses about the health of a past population is true. Most worryingly, they say this evidence is insufficient to determine whether past populations were healthy or not.

Yet, underdetermination alone is not unusual. Scientists commonly lack the data to arbitrate between competing hypotheses or theories, and it is rarely seen to pose a problem for progress. This is because we typically expect that underdeterminism will be resolved in the future as more evidence becomes available. Early in the novel coronavirus pandemic of 2020–21, for example, our theories about the virus itself were underdetermined by the evidence. There were multiple empirically adequate theories on the table concerning the transmission of the virus and the effectiveness of various public health interventions to reduce its spread. With time, however, we have built the body of evidence needed to rule out some theories and accept others. Underdetermination thwarted, progress is made.

### 5.1 Intractable Underdetermination

Things are not always so easy, however. Underdetermination can be intractable, particularly where further empirical investigation is hard to come by or unlikely to yield enough evidence to help us choose between competing theories or hypotheses. This type of underdetermination famously dogs cosmology—the study of the development, structure, history, and nature of the entire universe. There we see multiple cosmological models about the global structure of space time which are equally as compatible with our best observational data, and little reason to be optimistic that we will be able to get the sort of evidence required to arbitrate between them. Time and space are infinitely large, and we can explore neither directly beyond the micro‐local level. We can only access the global nature of the universe indirectly and via assumptions about the universality of the laws which apply to our bit of space time. For this reason, this type of cosmology is fiendishly epistemically hard, and some are pessimistic about its potential for progress (Smeenk 2013).

It is this sort of intractable underdetermination that the Osteological Paradox picks out. Wood et al.'s (1992) concern is not only that we do not have the type of skeletal evidence to decide between theories about past human health, but that we may never have sufficient evidence to do so. This sort of pessimism about the reliability of our access to the past is seen right across the historical sciences and related fields in the humanities such as history and biography. It has led some to the radical conclusion that we should be skeptical about the truth of all claims about the past (Hodder 1989; White 1980). The less extreme form of pessimism (which we focus on here) concerns the nature of evidence in the historical sciences and its limits.

### 5.2 The Pessimist's View

Pessimism of this type centers on two key concerns. First, the apparent paucity of the evidential base for historical sciences such as archaeology. Pessimists point out that the evidence of the past we can observe today, such as the skeletal record and other material traces, are at best patchy and incomplete. Only a small portion of the possible evidence of the past survives the ravages of time, and only a smaller portion of that is uncovered by the historical sciences. This means we can never be free of the possibility that the evidence we have is a biased or radically unrepresentative sample. This generates the sort of undetermination that should, say the pessimists, erode our trust in claims in the historical sciences (Sober 1988). A second concern derives from the apparent inaccessibility of history. Unlike in the case of the novel coronavirus, it is impossible to experiment on the past, or even to directly observe it. Thus, according to the pessimists, we can have “no new evidence” of the past. We can only hope to find the direct traces of historical events. In this, we are in a significantly weaker epistemic position than the epidemiologist or virologist studying a contemporary viral infection. To quote Derek Turner (2005, 227), “Although they can develop new technologies for identifying and studying potential smoking guns… [h]istorical scientists can never manufacture a smoking gun…”. The result of these two things is “the pessimistic view”; that some historical investigations will be “unlucky”, and some will be “lucky” depending on the vagaries of the information‐preserving and ‐destroying processes of time. When we have been unlucky, we should be pessimistic about slaying the dragon of underdetermination and thus scientific progress (Currie 2018). Wood et al.'s (1992) Osteological Paradox argues we are in the unlucky case when it comes to bioarchaeology. Although they point to some avenues for addressing the challenge—for example, that a better understanding of the pathological processes leading to bone lesions would result in less difficulty in interpreting bone lesions of past populations—the paradox has not been resolved.

### 5.3 Optimism (With Good Reason)

Yet, we see great cause for optimism. As Wood et al. (1992) pointed out in looking to pathology to constrain our hypotheses, we can produce new evidence via which they interpret the past by constructing controlled studies of physical and theoretical surrogates either through modeling or experimentation (Currie 2018). We can infer the types of tools used by past humans and the nature of their use by studying the microscopic differences in the cut marks made by tools in bone in the lab and then looking for these marks in ancient bones. These possibilities have now gone far beyond where Wood et al. (1992) could have expected with the development of more sophisticated microscopes and tools for 3D scanning, modeling, and analysis. Likewise, the value of agent‐based modeling for conducting controlled, experimental studies, with limitless iterations and variations, is abundantly clear.

In addition, carefully employed analogical reasoning (Chapman and Wylie 2016; Currie 2018)‐ drawing on parallels between similar cases to infer that regularities and causal patterns observed in one instance are likely to also be in play in another—allows us to infer connections between both different populations in the past, and past populations and contemporary ones, further bolstering our evidence. While there are perils to analogical reasoning, when part of a broader strategy which involves multiple lines of evidence and careful testing, it is a very fruitful source of evidence (Wylie 1985).

Which brings us to the means via which we can be assured our claims about the past are robust—by using multiple lines of evidence to support our claims (Chapman and Wylie 2016; Currie 2018; Wylie 1985). In the case of the Osteological Paradox, contemporary advances in DNA and epigenetic technologies, alongside digital experimentation such as agent‐based modeling, offer great opportunities here to add to traditional skeletal analyses and reduce ambiguity in our analyses.

Last, it is worth noting that the goal here is not to avoid uncertainty altogether. Rarely, if ever, in science do we work with certainties. Rather, the goal is to have evidence which allows us to arbitrate between hypotheses and theories sufficiently enough to adopt one over another. In this, inferences to the best explanation based on good evidential bases are enough. We do not need to avoid ambiguity or uncertainty at all to make progress. The realities of the historical sciences will always be with us. Our evidence of the past will always be patchy when compared to that of some other fields, but that does not entail that it is intractably so.

## 6 Conclusion

Since the publication of the Osteological Paradox, bioarchaeologists have sought ways to deal with the concerns it raised. Hidden heterogeneity and selective mortality are notoriously difficult to address, and many have resigned themselves to acknowledging and accepting the inferential limitations they present. Notwithstanding, some studies have engaged more directly with the Paradox—most notably through quantitative and statistical modeling. However, such strategies are not always feasible or accessible, particularly in cases where sample sizes are small, skeletal preservation is poor, or researchers lack access to specialized training or software. Even when statistics are employed, a critical and reflexive interpretive approach remains essential for responsible paleopathological analysis and bioarchaeological interpretation. While powerful, quantitative approaches are not without their own assumptions and limitations. Moreover, the majority of studies continue to rely on conceptual, descriptive, or methodological frameworks.

If we overlay the current conceptual and statistical approaches to the Osteological Paradox with an optimist's view of the historical sciences, our outlook is substantially improved. A syndemic approach, one that recognizes and integrates the multiple, intersecting (multi‐scalar and multidisciplinary) layers of information surrounding a skeletal population, can offer a productive path forward (Buikstra et al. 2022; DeWitte and Stojanowski 2015; Perry and Gowland 2022; Yaussy 2019) and is widely accepted in philosophy as a robust means of handling the uncertain nature of our past (Chapman and Wylie 2016; Currie 2018; Wylie 1985).

To this end, leveraging archaeological, environmental, and cultural contexts, particularly through interdisciplinary collaboration, can help minimize interpretive biases. The integration of historical, social, political, environmental (e.g., climate, pollution, urbanization), and archaeological information enhances our ability to interpret skeletal data with greater nuance and caution. Isotope analysis, ancient DNA, paleoproteomics, and archaeotoxicology, can contextualize skeletal evidence and refine our understanding of individual life histories and health risks. This kind of interdisciplinary engagement, a hallmark of bioarchaeology, provides essential contextual depth and strengthens interpretations in the face of the challenges posed by the Paradox. At the biological level, focusing on non‐adults as non‐survivors, and incorporating age‐structured analyses of morbidity, provides another promising strategy to explore frailty and survivorship within a framework sensitive to the Paradox. Lastly, ongoing research into the etiology and physiological mechanisms behind skeletal lesion formation, and their associations with frailty or disease susceptibility (e.g., van Schaik et al. 2014; O'Donnell et al. 2023; McFadden and Oxenham 2020; Anderson et al. 2025)—an example of controlled experimentation and analogical reasoning (Chapman and Wylie 2016; Currie 2018)—continues to inform and refine how we interpret these markers in the archaeological record. A reflexive, interdisciplinary, and methodologically transparent approach remains essential for producing interpretations that are both scientifically grounded and intellectually honest.

Beyond applied bioarchaeological research, agent‐based modeling provides an experimental space for the Paradox to be further explored and evaluated without the constraints of the historical and pre‐historical sciences. As bioarchaeologists move towards quantifying the sources of compounding uncertainty that confound interpretations of health from the skeletal record, the Osteological Paradox can be mapped and measured, and many of the frequently discussed unknowns can be identified, quantified, and dealt with. Addressing these persistent sticking points, we believe, will facilitate substantial evaluation and progress within the field. This exciting new chapter of bioarchaeology has already started (see Wyatt et al. 2025) and holds enormous potential for applications to the Paradox's concerns.

Finally, we believe a shift in perspective is both warranted and essential to progressive treatment of the Osteological Paradox. The Paradox's concerns have long loomed over bioarchaeological research as somewhat of a gray cloud. But these problems are far from unique to the Paradox, nor the discipline, and there is much to be learned from how underdetermination is dealt with in other academic fields. A shift away from unrealistic expectations of certainty is necessary to allow the discipline to continue to flourish, while in parallel new physical and digital technologies afford new opportunities for multi‐line evidence, controlled experimentation, and convergent inferences.

Our workshop brought together diversely skilled individuals, and that is clearly reflected in this synthetic review. One of the things we find striking is that, considering these varying perspectives collectively, we have arrived at a standpoint that values the contribution of past research and solidifies its ongoing importance and paves a new, parallel and interweaving path for forthcoming research. We hope that this review will encourage current scholars to rethink their relationship with the Osteological Paradox. The future of the discipline is bright. With an optimist's outlook, an arsenal of conceptual and analytical tools, numerous opportunities for interdisciplinary collaboration, and the exciting opportunities that agent‐based modeling presents, there exists a plethora of delicious low‐hanging fruit ripe for the picking. Go forth and harvest!

## Author Contributions

**Clare McFadden:** conceptualization, writing – original draft, writing – review and editing, investigation, project administration, methodology. **Rachael Brown:** conceptualization, writing – original draft, investigation, methodology, writing – review and editing. **Bronwyn Wyatt:** conceptualization, investigation, writing – original draft, methodology, writing – review and editing. **Sammuel Sammut:** conceptualization, investigation, writing – original draft, methodology, writing – review and editing. **Kathryn E. Marklein:** conceptualization, investigation, writing – original draft, methodology, writing – review and editing. **Samantha L. Yaussy:** conceptualization, investigation, writing – original draft, methodology, writing – review and editing. **Amy Anderson:** conceptualization, investigation, writing – original draft, writing – review and editing, methodology. **Lucie Biehler‐Gomez:** writing – review and editing, conceptualization, investigation, writing – original draft, methodology.

## Funding

This work was supported by the National Geographic Society (EC‐67820R‐20), Australian National University.

## Data Availability

Data sharing not applicable to this article as no datasets were generated or analysed during the current study.
